# De novo transcriptome and tissue specific expression analysis of genes associated with biosynthesis of secondary metabolites in *Operculina turpethum* (L.)

**DOI:** 10.1038/s41598-021-01906-y

**Published:** 2021-11-18

**Authors:** Bhagyashree Biswal, Biswajit Jena, Alok Kumar Giri, Laxmikanta Acharya

**Affiliations:** Molecular Biology and Genetic Engineering Lab, Centre for Biotechnology, School of Pharmaceutical Sciences, Siksha ‘O’ Anusandhan (Deemed to be University), Bhubaneswar, Odisha India

**Keywords:** Biotechnology, Transcriptomics

## Abstract

This study reported the first-ever de novo transcriptome analysis of *Operculina turpethum,* a high valued endangered medicinal plant, using the Illumina HiSeq 2500 platform. The de novo assembly generated a total of 64,259 unigenes and 20,870 CDS (coding sequence) with a mean length of 449 bp and 571 bp respectively. Further, 20,218 and 16,458 unigenes showed significant similarity with identified proteins of NR (non-redundant) and UniProt database respectively. The homology search carried out against publicly available database found the best match with *Ipomoea nil* sequences (82.6%). The KEGG (Kyoto Encyclopedia of Genes and Genomes) pathway analysis identified 6538 unigenes functionally assigned to 378 modules with phenylpropanoid biosynthesis pathway as the most enriched among the secondary metabolite biosynthesis pathway followed by terpenoid biosynthesis. A total of 17,444 DEGs were identified among which majority of the DEGs (Differentially Expressed Gene) involved in secondary metabolite biosynthesis were found to be significantly upregulated in stem as compared to root tissues. The qRT-PCR validation of 9 unigenes involved in phenylpropanoid and terpenoid biosynthesis also showed a similar expression pattern. This finding suggests that stem tissues, rather than root tissues, could be used to prevent uprooting of *O. turpethum* in the wild, paving the way for the plant's effective conservation. Moreover, the study formed a valuable repository of genetic information which will provide a baseline for further molecular research.

## Introduction

The pantropical genus *Operculina* is a major taxon of the family Convolvulaceae comprising 15 species. *Operculina turpethum* (L.) Silva Manso, popularly known as Indian jalap/ Turpeth/ Nisoth/ Trivrit, is one of the most industrially and therapeutically important medicinal herb of the morning glory family. This perennial vine is native to the temperate and tropical region of Asia (India, Nepal, Bangladesh, Pakistan, Sri Lanka, China, Taiwan, and Myanmar). It is also found to grow in some parts of Australia, Africa (Somalia, Kenya, Tanzania, Zimbabwe, Mozambique, Comoros, Madagascar, and Mauritius), Pacific Islands and southern America (West Indies). The plant is commonly found in the moist deciduous and tropical dry region of peninsular and central India (Western Ghats, Kerala, Karnataka, and Tamil Nadu). *O. turpethum* root and stem are key ingredients in more than 135 herbal formulations in both Unani and Ayurvedic medicine system which are used to treat diverse ailments including obesity, constipation, gastric ulcer, diarrhea, asthma, uterine problem, cough splenomegaly, jaundice, anemia, hyperlipidemia, tumors, joint and muscle pain, paralysis and rheumatoid arthritis and tuberculosis. In addition, extensive pharmacological studies of different extracts of *O. turpethum* with animal models have demonstrated the antibacterial^1,2^, analgesic^3^, antioxidant^4^, anti-inflammatory, anti-cancer^5^, anti-diabetic^6^, hepato-protective^7^, anti-ulcer^8^, anti-arthritic^9^, immune-modulatory^10^ and anti-nephrotoxic, antispasmodic, bronchodilator^11^, laxative^12^, and larvicidal potential^13^. The therapeutic potential is ascribed to their bioactive constituents which includes flavonoids, coumarin, scopoletin, Coumaric acid derivatives (N-p-coumaryl tyramine), triterpenoid (lanosta‑5‑ene, cycloartenol and 24‑methylene‑δ‑5‑lanosterol) dammarane type triterpenoid saponin (operculinosides A, B, C, D)^14^, resin glycoside (turpethosides A, B)^15^, glycosidic acid (turpethic acids A–C), acrylamide, phytosterol (daucosterol and β-sitosterol), betulin, lupeol, α‑ and β‑turpethein, and steroid glycoside, etc. Previous phytochemical studies of *O. turpethum* showed that the oxygen containing monoterpenes such as carvacrol, caryophyllene oxide and Thymol were reported to be the major compounds in the volatile oil^16^. Another important bioactive compound Stigma-5,22dien-3-O-β-D-glucopyranoside, a steroidal glycoside with anticancer, immune-stimulatory, hepatoprotective, antioxidant activity was also isolated from *O. turpethum*^17–19^. Apart from the pharmacological application, the seeds of the plant are reported to be a potential source of commercial gum^20^. However excessive exploitation of root/root bark over the other part of the plant has caused depletion of its germplasm resources from the wild while bringing it to Near Threatened category^21^ which subsequently leads to unavailability of the requisite plant material followed by adulteration.

Despite the fact that O. turpethum has been extensively studied in the phytochemical and pharmacological fields, no reports on transcriptomic and genetic studies of the plant are available in the public database, which are required for elucidating the metabolic pathways of the active ingredients, improvement of germplasm as well as the study of genetic diversity and proper identification of the plant.

The advent of high throughput next generation sequencing platform has dramatically reformed the perception of the complex and dynamic character of transcriptome by providing better comprehensive and quantitative aspects of gene expression, allele specific expression, alternative splicing, gene fusion and detection of functional gene. In addition, the high throughput RNA-Sequencing (RNA-Seq.) approach has been widely employed for the development of molecular markers such as Single nucleotide polymorphism” (SNPs) and “Simple sequence repeats (SSRs)”, which can play a significant role in the study of medicinal plant phylogeny and genetic diversity^22,23^. Besides, the de novo transcriptome approach is a predominantly used cost-effective technique which allow us to identify all expressed transcripts in non-model plants lacking good-quality reference genome information. Currently, the Illumina NGS platform has become the main workhouse for the generation of massive amounts of sequence information with respect to genomics and transcriptomics data for various non-model plant and animal species.

In the present study the transcriptome sequencing of only root and stem tissues of *O. turpethum* was carried out as both these tissues have been reported to contain several bioactive phytochemicals which account for the tremendous pharmacological activities of the potent medicinal plant. The sequence data obtained from the Illumina 2 × 150 paired-end platform were assembled and annotated in multiple databases and the genes related to secondary metabolite biosynthesis were identified. All together the transcriptome data will serve as a foundation to explore the plant at the genomic and transcriptomic levels.

## Result and discussion

### RNA sequencing and assembly

**Figure 1:**
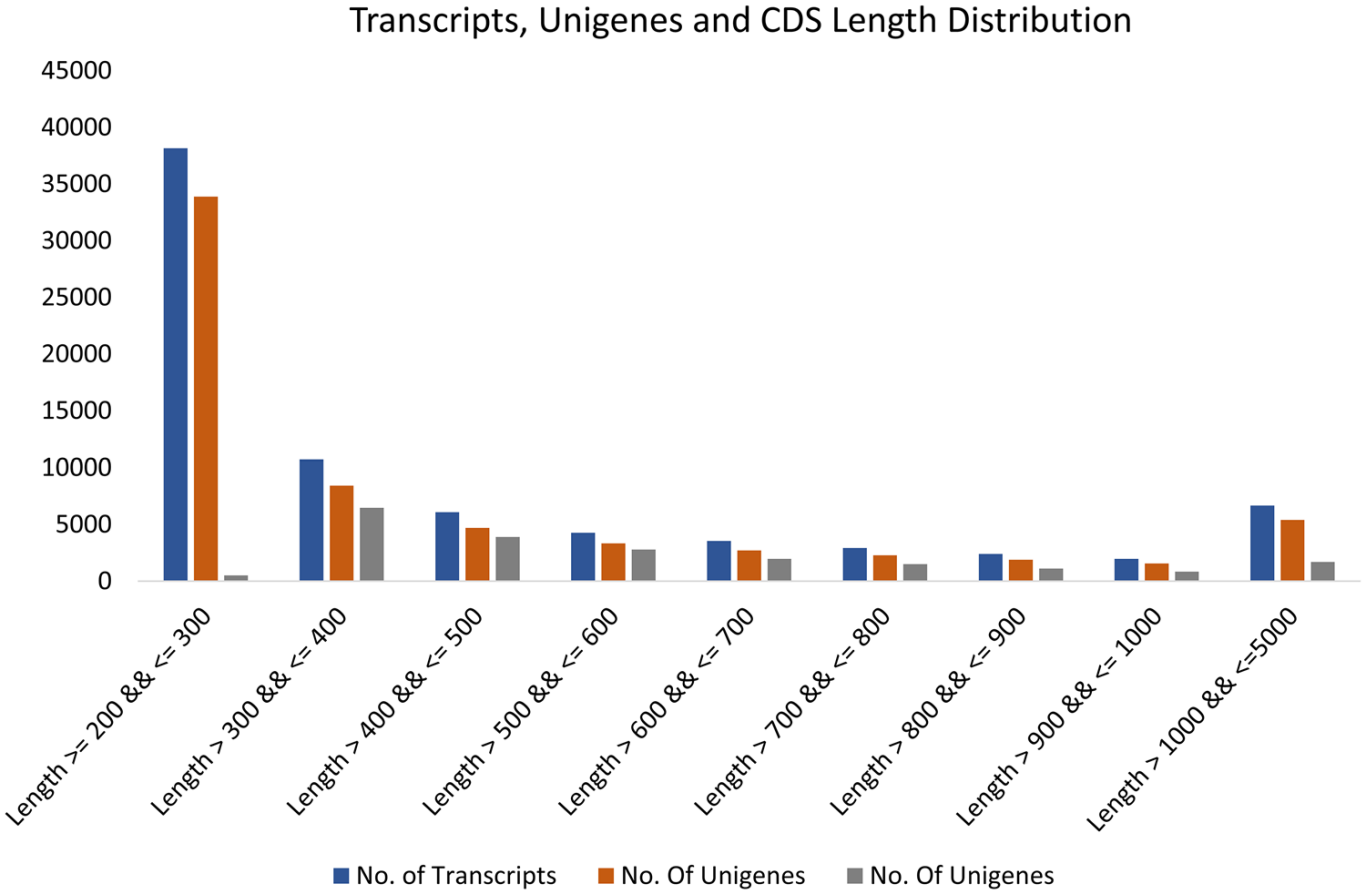
Transcripts, unigenes and CDS length distribution.

The libraries sequenced using 2 × 150 bp PE chemistry on the Illumina platform generated ~ 5 GB of data per sample. After filtering, a total of 28,622,974 (root) and 26,898,420 (stem) raw reads were obtained having a Q20 base value (base quality more than 20) of 96.83%. A de novo assembly was done as no reference genome data was available for *O. turpethum.* Master assembly was performed taking reads of Root and Stem samples together using Trinity (at default parameters, k-mer 25). The raw reads upon assembling, a total of 76,790 transcripts for both root and stem taken together. The total transcript size was 35,332,145 bp with an average transcript length of 460 bp. The maximum transcript length was 4104 bp and a N50 length of 583 bp (Table 1). CD-HIT-EST executable was used to eliminate the shorter redundant sequences which have more than 90% identity with 100% coverage for any other transcripts and the clustered non-redundant transcripts thus obtained were designated as unigenes. This was done as low-quality bases and the presence of adapters in reads may hamper the assembly process resulting in mis assembly or truncated contigs. The total number of Unigenes obtained for both root and stem was 64,259 with a total of 28,856,611 bases in unigenes. The mean unigene length was 449 bp with a maximum length of 4104 bp and N50 length of 564 bp. (Table 1), where the length of majority of unigenes ranges from 200 to 500 bp (Fig. 1, Supplementary Table S1). The higher N50 value further approves of a better-quality assembly. The CDS prediction was done from these unigenes using Transdecoder at default parameters a minimum length of 100 amino acids of the encoded protein plus homology search with Pfam and UniProt databases. A total of 20,870 CDS were obtained having a total of 11,929,458 bases. The mean CDS length tends to be 571 bp with a maximum length of 2745 bp. The maximum number of CDS belonged to 300 to 400 bp length and minimum to the account of 200 to less than 300 bp. (Fig. 1, Supplementary Table S2).

**Table 1:** Statistics of assembled transcripts and unigenes.

Description	Number of transcripts	Number of unigenes
Length ≥ 200 && ≤ 500 bp	54,979	47,043
Length ≥ 500 && ≤ 1000 bp	15,136	11,804
Length ≥ 1000 && ≤ 5000 bp	6675	5412
Total no	76,790	64,259
Total number of bases (bp)	35,332,145	28,856,611
Average length (bp)	460	449
Maximum length	4104	4104
N50 (bp)	583	564

### Homology search and functional annotation

**Figure 2:**
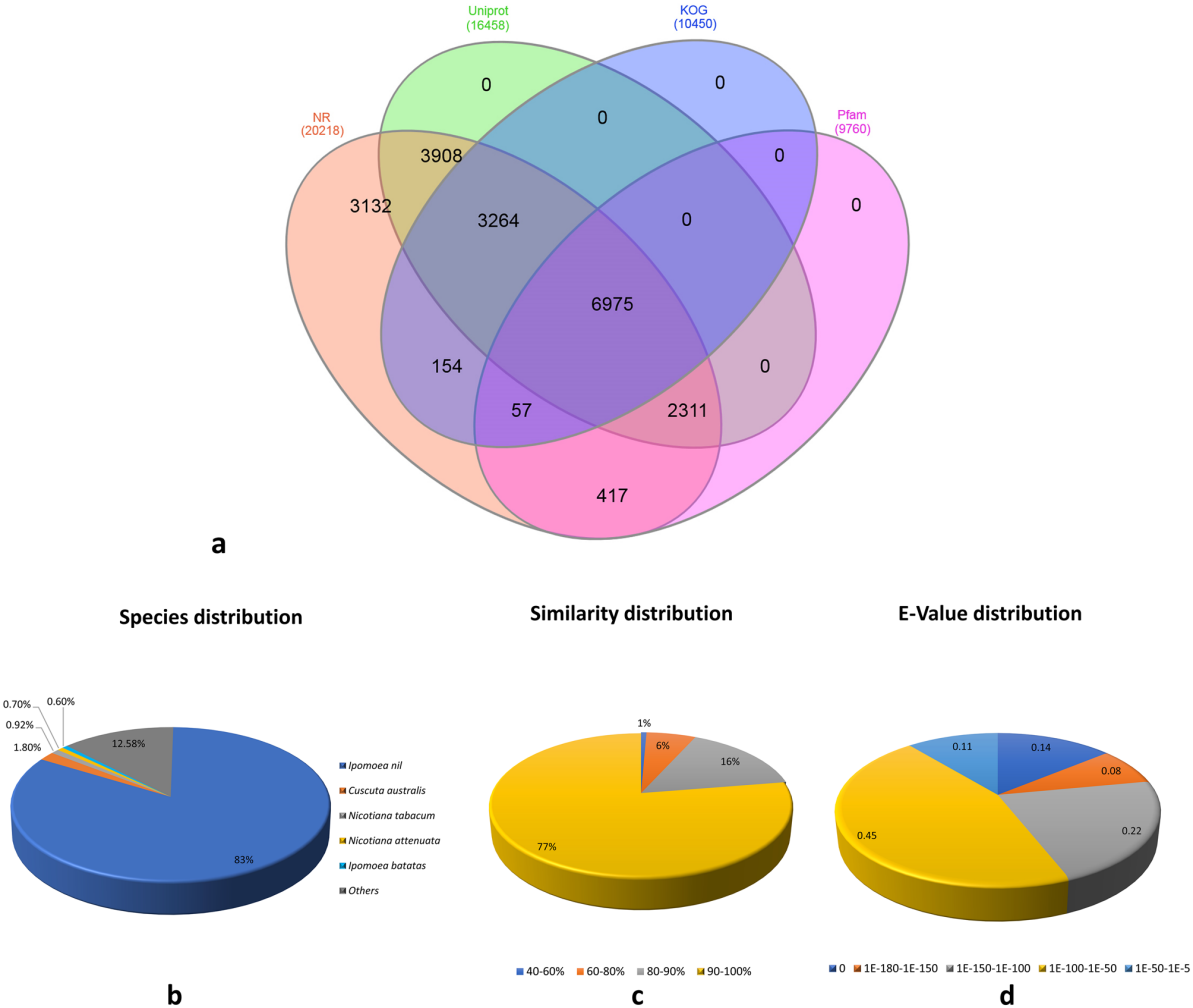
(**a**) Venn diagram for Root-Stem annotated Unigenes in different databases, (**b**) Species Distribution**,** (**c**) E-value distribution and (**d**) Similarity distribution.

A total of 20,870 unigenes (32.5%) out of 64,259 assembled unigenes were annotated functionally by searching against NCBI non-redundant (Nr) protein sequence database, UniProt, Clusters of Orthologous group of protein (KOG/COG), and Pfam database using BLASTX with an E-value threshold of 1E^−5^. On doing the similarity search, it was found that 20,218 unigenes could be annotated to the Nr database whereas, 16,458 unigenes had similarity with UniPort, 10,450 with KOG, and 9760 with Pfam database. The comparative count of unigenes annotation in different databases was depicted in the form of a Venn diagram, which showed that a total of 6975 unigenes were co-annotated in four databases (Fig. 2a).

The top-hit species distribution analysis revealed that most of the *O. turpethum* unigenes (16,717, 82.6%) had significant homology with *Ipomea nil* sequences followed by *Cuscuta australis* (374, 1.8%) (Fig. 2b). This was the case because both the plant species, *O. turpethum and I. nil* belong to the Convolvulaceae family and morphologically look quite similar to each other except the flower color and size, whereas *Cuscuta australis* although belonged to the same family showed significant morphological difference and belonged to plant parasite group. Furthermore, the E-value distribution of the top-hits showed that 89% of the mapped sequences had significantly high scores for homology (E-value < 10^−50^), whereas 11% of the annotated sequences exhibited homology with e-value ranging from E^−5^ to E^−50^ (Fig. 2c). Likewise, around 93% of the annotated sequences were found to have similarities above 80% (Fig. 2d). These outcomes indicate the high similarity of the annotated sequences with the known sequences available in the public database, implying good quality assembly. However, there exists a large number of sequences (43,389, 67.52%) without any BLAST hits which may be due to the presence of new/novel genes performing function related to specific plants or due to the presence of untranslated regions or the short sequence lacking the conserved protein domain.

**Figure 3:**
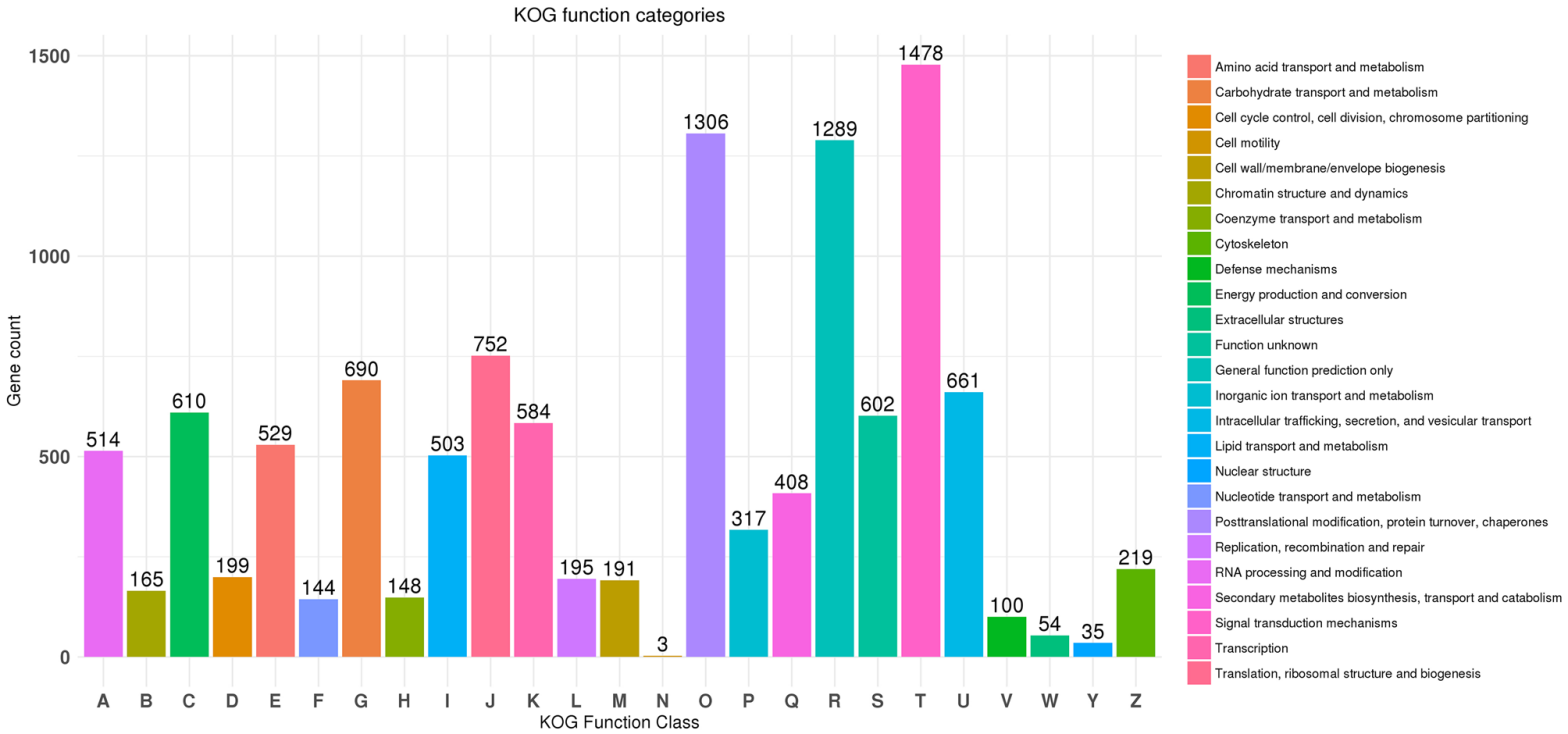
KOG classification for Root-Stem CDS.

KOG classification produced hits for 10,450 unigenes which were further classified into 25 KOG functional categories (Fig. 3). The highly enriched KOG category for the Root-Stem sample was “Signal transduction mechanisms (T)” with 1478 unigenes followed by “Posttranslational modification, protein turnover, chaperones (O)” (1306) and General function prediction only (R)” (1289). In Pfam analysis, the most abundant domains identified were representing “Pkinase” with 310 unigenes followed by “Pkinase_Tyr” (248) and “P450” (131). The top 10 most abundant Pfam domains and their counts were shown in the table below (Table 2).

**Table 2:** Top Pfam domain in Root-Stem.

Domain	Count
Pkinase	310
Pkinase_Tyr	248
p450	131
RRM_1	128
zf-RING_2	83
Ras	77
COX1	75
PP2C	71
WRKY	65

### GO classification of NR annotated unigenes

**Figure 4:**
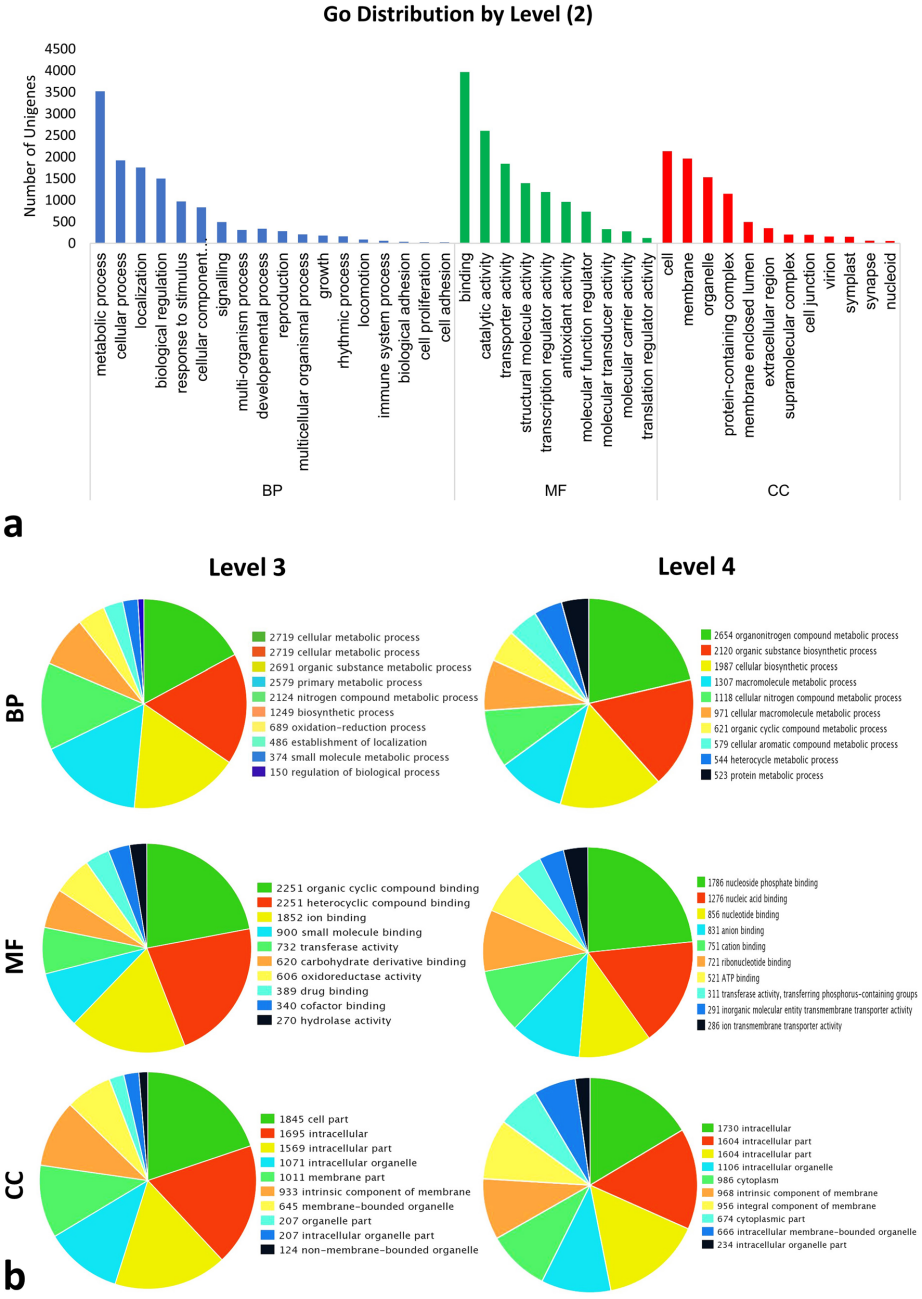
Gene Ontology distribution for Root-Stem sample. (**a**) level 2, (**b**) level 3 and 4. The Unigenes were assigned to three main categories: *BP* Biological processes, *MF* Molecular functions, *CC* Cellular components.

Out of 20,218 NR annotated unigenes, a total of 10,991 unigenes were assigned one or more than one GO terms The GO classification system comprises 3 main domains: Biological process (BP: 3824 unigenes), cellular component (CC:2632 unigenes), and Molecular function (MF:5216 unigenes), which were further divided into 40 subcategories in level 2 GO term annotation. . Among biological processes, metabolic process (27.7%) accounts for the largest proportion followed by cellular process (15.1%), localization (13.8%) and biological regulation (11.8%). Under the cellular component domain, cell (25.3%) followed by plasma membrane (23.2%) were the most enriched subcategories. In molecular function, binding (29.6%) was the highly represented category followed by catalytic activity (19.4%) and transporter activity (13.73%) (Fig. 4a).

Each of the three GO domain was further categorized into level 3 and level 4 GO terms for the extensive function analysis basing on GO database. For example, Binding was the most enriched level 2 term for molecular function and in the level 3 term, the binding activity was assigned to some specific function such as ‘heterocyclic compound binding’ (GO:1901363, 2251 unigenes), ‘organic cyclic compound binding’ (GO:0097159, 2251 unigenes), ‘ion binding’ (GO:0043167, 1852 unigenes) (Fig. 4b). Furthermore, in level 4 term, the organic cyclic compound binding (belonging to binding activity) was subcategorized into ‘nucleoside phosphate binding’ (GO:1901265, 1786 unigenes), ‘nucleic acid binding’ (GO:0003676, 1276 unigenes), ‘nucleotide binding’ (GO:0000166, 856 unigenes), ribonucleotide binding (GO:0032553, 721 unigenes), (Fig. 4b).

### Pathway analysis using KEGG

Among the sequences examined against KEGG database, 6518 unigenes were functionally assigned to 378 KEGG modules belonging to five main pathway categories, of which Metabolism was the most abundant category with 2751 unigenes (42.20%) followed by genetic information processing (1466 unigenes, 22.49%), environmental information processing (963 unigenes, 14.7%), cellular process (944 unigenes, 14.4%) and organismal system (394 unigenes, 6.02%)^24–26^. Among metabolism, most of the unigenes were involved in carbohydrate metabolism (21.7%), and amino acid metabolism (14.6%) followed by lipid metabolism (12.4%), energy metabolism (10.5%), and biosynthesis of other secondary metabolites (10.5%) (Supplementary Table S3). Supplementary Fig. S1a represents the distribution of top 20 most enriched pathways according to KEGG database with Signal transduction” as the most abundant pathway comprising of 946 unigenes followed by “Translation” (602) and “Carbohydrate metabolism” (595), “Transport and catabolism” (527). In this study, the unigenes involved in Terpenoid and polyketide metabolism and other secondary metabolite biosynthesis were detected which support the presence of diverse secondary metabolites in *O. turpethum* (Supplementary Fig. S1b). Along with the above data, this study also identified some of the biosynthesis pathways related to antimicrobial compounds including Streptomycin biosynthesis (PATH:ko005210), Neomycin, kanamycin and gentamicin biosynthesis (PATH:ko00524), Novobiocin biosynthesis (PATH:ko00401), which were also reported to be present in *Phyllanthus amarus* and *Plumbago zeylanica* transcriptomes^27,28^. The functional characterization of the non-model plant *O. turpethum* revealed that the de novo transcriptome analysis based on RNA-seq will promote further research on the biochemistry, molecular genetics, and physiology of *O. turpethum* or related species.

### Differential gene expression analysis in root versus stem sample

**Figure 5:**
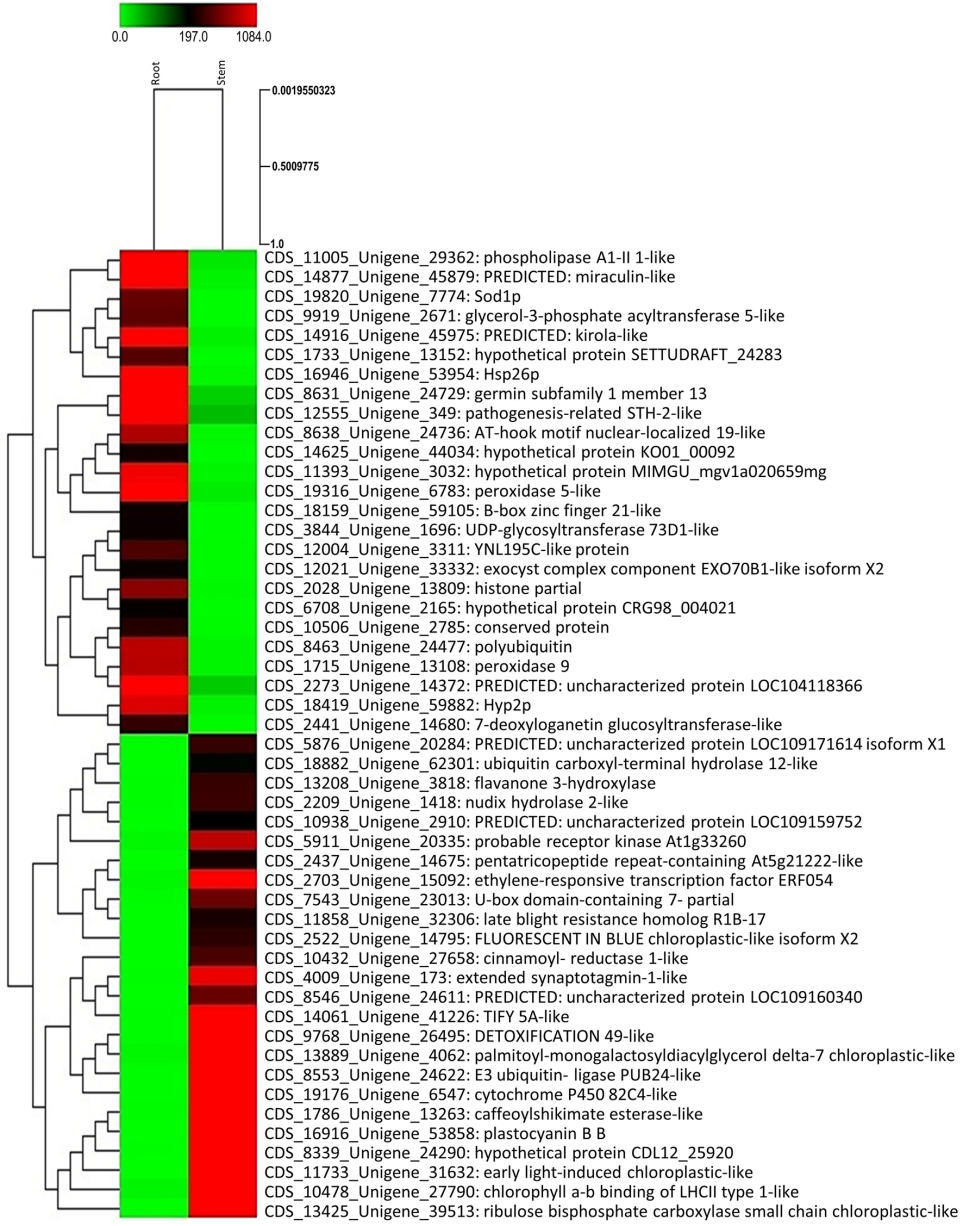
Heat map representing top 50 Highly upregulated and highly downregulated unigenes in Root-versus-Stem.

Overall, 17,444 DEGs (Differentially expressed genes) were identified out of which 8722 genes were upregulated and 8722 genes were downregulated in root versus stem system (Supplementary Table S4). The statistical analysis of the expression of tissue specific unigenes revealed that 451 and 2975 unigenes were exclusively expressed in the root and stem tissues of *O. turpethum* respectively. The Scatter Plot and Volcano Plot (Supplementary Fig. S2) represent the upregulated and downregulated unigenes in root and stem tissues. Additionally, the hierarchical clustering approach was used to represent the top 50 highly upregulated and highly downregulated genes in the form of heatmap (Fig. 5).

### Identification of genes involved in phenylpropanoid biosynthesis in *O. turpethum*

In the present study, it was found that the phenylpropanoid biosynthesis pathway contained a maximum number of unigenes (190) encoding 16 key enzymes (Supplementary Table S5) associated with phenylpropanoid biosynthesis (PATH: ko00940) (Supplementary Fig. S3)^24–26^ and hence taken into further consideration. Phenylpropanoids are the diverse class of natural products whose biosynthesis is known to begin from the deamination of an aromatic amino acid Phenyl alanine by Phenyl alanine ammonia lyase (PAL, EC:4.3.1.24, 6 unigenes) to form Cinnamic acid which is then hydroxylated by cinnamate-4-hydroxylase (EC:1.14.14.91, 1unigene) to form *p*-coumaric acid. *The* addition of a CoA thioester to *p*-coumaric acid by the enzyme 4-coumarate-Coenzyme A ligase (EC: 6.2.1.12, 8 unigenes) enzyme gives rise to *p*-coumaroyl CoA which serves as high energy intermediate in the biosynthesis of lignin (cell wall component), flavonoids (pigments), pest resistance and UV protected compound (Isoflavonoids, flavonoids, stilbenes, furanocoumarins, and coumarins)^29^. The digital gene expression analysis revealed that gene encoding enzymes such as PAL (6 unigenes), CYP73A (1 unigene), 4CL (5 unigenes), HCT (2 unigenes), C3'H (1 unigene), COMT (1 unigene), F6H, involved in the biosynthesis of Scopoletin (coumarin), were found to be significantly upregulated in stem tissues with some of them showing stem specific expression. Phenylpropanoids are reported to support plant growth and survival by providing protection against UV- radiation and photo-oxidative effect, strengthening specialized cell wall thereby providing vascular integrity, structural support, and pathogen resistance to plants and stimulation of symbiotic nitrogen fixation^30^. Furthermore, the phenylpropanoids and their derivatives were also known to possess several biological activities including antioxidant, antimicrobial, anticancer, antidiabetic, neuroprotective activity. The compounds also exhibit significant application in food and cosmetics industry due to their antimicrobial, antioxidant, and photoprotective activity^31^.

### Identification of unigenes involved in flavonoid biosynthetic pathway

The Flavonoids (flavonols, flavandiols, flavones, chalcones, anthocyanins) are synthesized via phenylpropanoid biosynthesis pathway and are known to be accountable for the coloration of flowers, fruits and seeds, plant reproduction and fertility, auxin transport, nodulation and also involved in defense mechanism by protecting the plant against UV-radiation, pathogen infection, herbivore attack, metal toxicity, etc.^32^ Interestingly, the present study identified 17 unigenes encoding 9 key enzymes (Supplementary Table S6) involved in flavonoid biosynthesis (PATH: ko00941)^24–26^ (Supplementary Fig. S4). Chalcone synthase (EC:2.3.1.74, 2 unigenes), the first enzyme specific for flavonoid biosynthesis pathway which converts 4-coumaryl CoA to Chalcone, was found to be upregulated in the stem tissues based on the digital gene expression analysis. The isomerization of Chalcone to Naringenin is catalyzed by the enzyme Chalcone isomerase (EC:5.5.1.6, 3 unigenes) which was also found to be highly expressed in stem tissues (18 folds) as compared with root tissues. Naringenin then enters into the late step of flavonoid biosynthesis from which all other flavonoids are derived. Again, the pathway annotation revealed that the unigenes encoding anthocyanidin 3-O-glucosyltransferase (EC:2.4.1.115) and kaempferol 3-O-beta-D-galactosyltransferase (EC:2.4.1.234), were found to be stem specific transcripts which are known to be involved in anthocyanin and flavonol glycoside biosynthesis respectively.

Previous studies have reported the presence of Flavonoids like quercetin, kaempferol, and the flavonoid glycoside, Formononetin 7-O-*β*-D-glucopyranoside in the aerial parts of *O. turpethum,* which also reported to exhibit anti-arthritic, immunomodulatory, anti-oxidant, and anti-inflammatory activity^9,10^. From the comparative gene expression analysis, it was found that most of the unigenes encoding CHS, CHI, F3H, FLS, DFR, BZ1, CYP81E were highly expressed in stem tissues indicating that they might be the key gene in regulating the biosynthesis of flavonoids which require further functional characterization.

### Identification of key genes involved in terpenoid biosynthesis pathway

Similarly, terpenoids comprise the largest group of structurally diverse natural compounds and are known to biosynthesize via two routes: ‘2-C-methyl-D-erythritol 4-phosphate (MEP)’ pathway and ‘mevalonate acid (MVA)’ pathway^33^. The isoprene unit (C_5_) synthesized from the MEP pathway is engaged in the formation of mono-(C10), Di-(C20), and some polyterpenoids^34^ whereas the isoprene unit from the MVA pathway is used in the synthesis of triterpene (C30) and Sesquiterpene (C15)^35^. In the dataset, 86 genes were found to encode 42 key enzymes (Supplementary Table S7). The identified 43 unigenes of terpenoid backbone biosynthesis (PATH: ko00900, 43 unigenes)^24–26^ (Supplementary Fig. S5) encoded 7 enzymes for each of the MEP and MVA pathway and the MEP pathway-related gene showed high expression in stem tissues as compared with the root tissue. 4 unigenes were found to be involved in monoterpenoid biosynthesis (PATH: ko00902)^24–26^ (Supplementary Fig. S6) encoding Neomenthol dehydrogenase (EC: 1.1.1.208, 1unigene) and 8-Hydroxygeraniol dehydrogenase (EC: 1.1.1.324, 3 unigenes) and 7 unigenes were predicted to be associated with diterpenoid biosynthesis (PATH: ko00904)^24–26^ (Supplementary Fig. S7) including ent-kaurene synthase (EC: 4.2.3.19, 1 unigene), ent-kaurene oxidase (EC: 1.14.14.86, 1 unigene), gibberellin-44 dioxygenase (EC: 1.14.11.12, 1 unigenes), gibberellin 2beta-dioxygenase (EC: 1.14.11.13, 3 unigenes) and trimethyltridecatetraene/dimethylnonatriene synthase (EC: 1.14.14.58/1.14.14.59). Furthermore, 7 unigenes were identified as Sesquiterpenoid and triterpenoid biosynthesis (PATH: ko00909)^24–26^ (Supplementary Fig. S8) related gene encoding Squalene synthase (farnesyl-diphosphate farnesyltransferase) (EC:2.5.1.21, 2 unigenes), squalene monooxygenase (EC: 1.14.14.17, 1 unigene), NAD+-dependent farnesol dehydrogenase (EC: 1.1.1.354, 2 unigenes), (3S,6E)-nerolidol synthase (EC: 4.2.3.48, 1 unigene) and germacrene D synthase (EC: 4.2.3.75, 1 unigene). According to the digital gene expression analysis, the one unigene of each of isoprene synthase (CDS_3411_Unigene_16235), prenyl protein peptidase (CDS_11648_Unigene_3123), NAD+-dependent farnesol dehydrogenase (CDS_5038_Unigene_19017), Squalene synthase (CDS_13671_Unigene_4006), and (3S,6E)-nerolidol synthase (CDS_7899_Unigene_23587) were found to be exclusively present in stem tissues of *O. turpethum.* The differential expression patterns of the genes may be responsible for the differential accumulation of previously reported terpenoids such as Carvacrol, Thymol, Cycloartenol, Lanosta-5-ene, 24-methylene-δ-5-lanosterol, lupeol, betulin, linalool in *O. turpethum.*

### qRT-PCR validation of gene expression profiling

**Figure 6:**
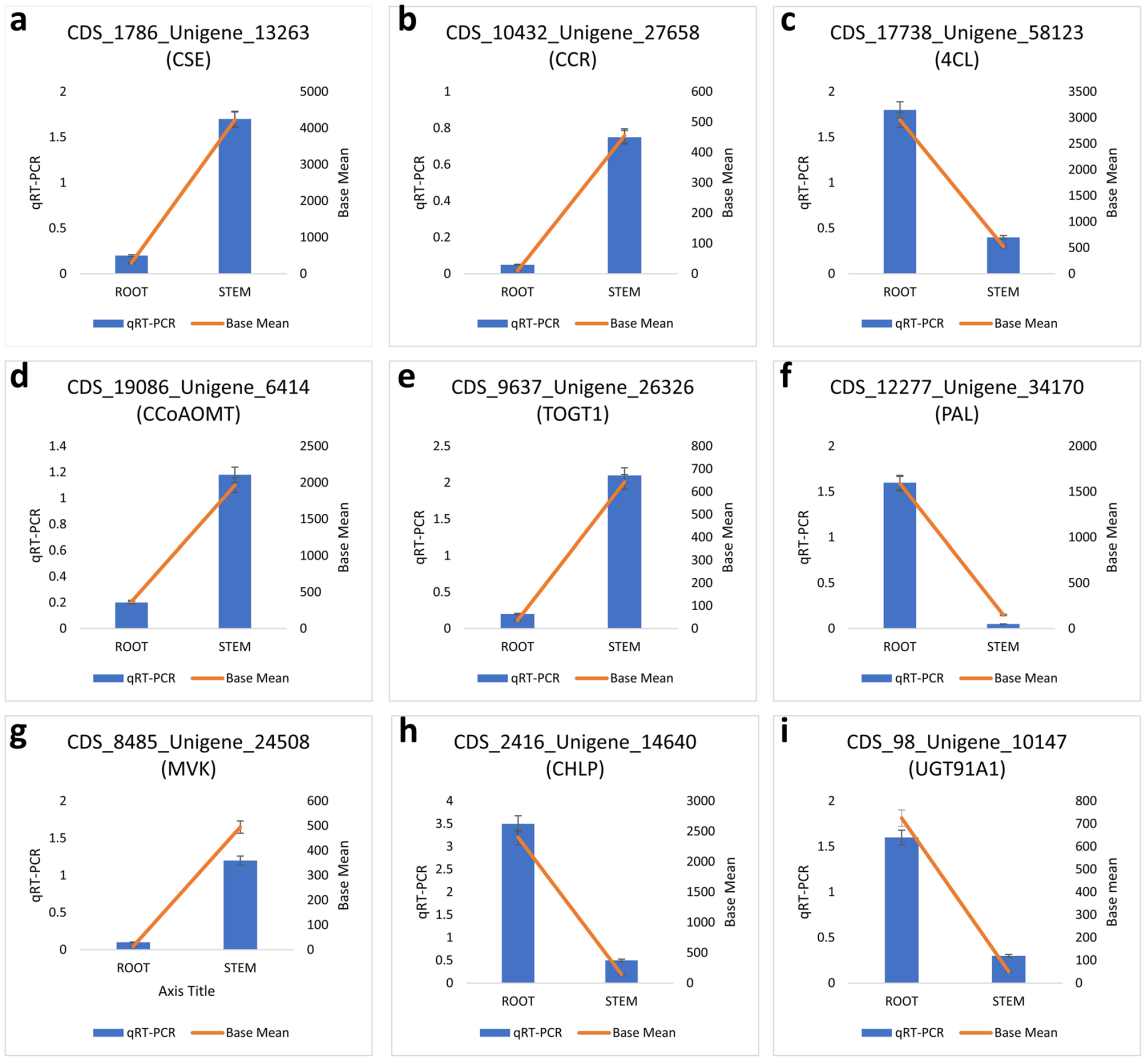
The qRT-PCR analysis of nine candidate unigenes encoding enzymes involved in phenylpropanoid and terpenoid biosynthesis in root and stem of *O. turpethum*. Blue bars indicate the qRT-PCR results and red lines show the base mean values identified via the RNA-Seq analysis. Data is shown as the mean ± standard error of mean of three replicates. The left y-axis is the relative expression level of unigenes obtained by qRT-PCR and the right y-axis denotes the base mean values in the RNA-Seq data.

Currently, qRT-PCR is an important tool for determining gene expression level because of its sensitivity, specificity, reproducibility and accuracy. Moreover, it has become the method of choice to validate the gene expression profiling data obtained from large scale transcriptome research^27,28^. Nine unigenes involved in the phenylpropanoid and terpenoid biosynthesis were selected for the qRT-PCR experiment to validate their differential expression pattern between the root and stem tissues of *O. turpethum*. The relative expression of unigenes encoding Caffeoyl shikimate esterase, Cinnamoyl- reductase 1, Caffeoyl- O-methyltransferase, Scopoletin glucosyltransferase, and Mevalonate kinase were found to be higher in the stem tissues whereas unigenes encoding 4-coumarate-ligase 2, phenylalanine ammonia-lyase G, Geranylgeranyl diphosphate, UDP-glycosyltransferase, showed higher expression in the root tissues of *O. turpethum.* All the selected unigenes showed an expression pattern similar to those obtained from the Digital gene expression analysis (Pearson correlation coefficient = 0.89), indicating the high reliability of the RNA-Seq data. The results were shown in Fig. 6. The experimentally validated gene expression data will provide a better insight of function and regulation of genes.

### Identification of transcription factors (TFs)

**Figure 7:**
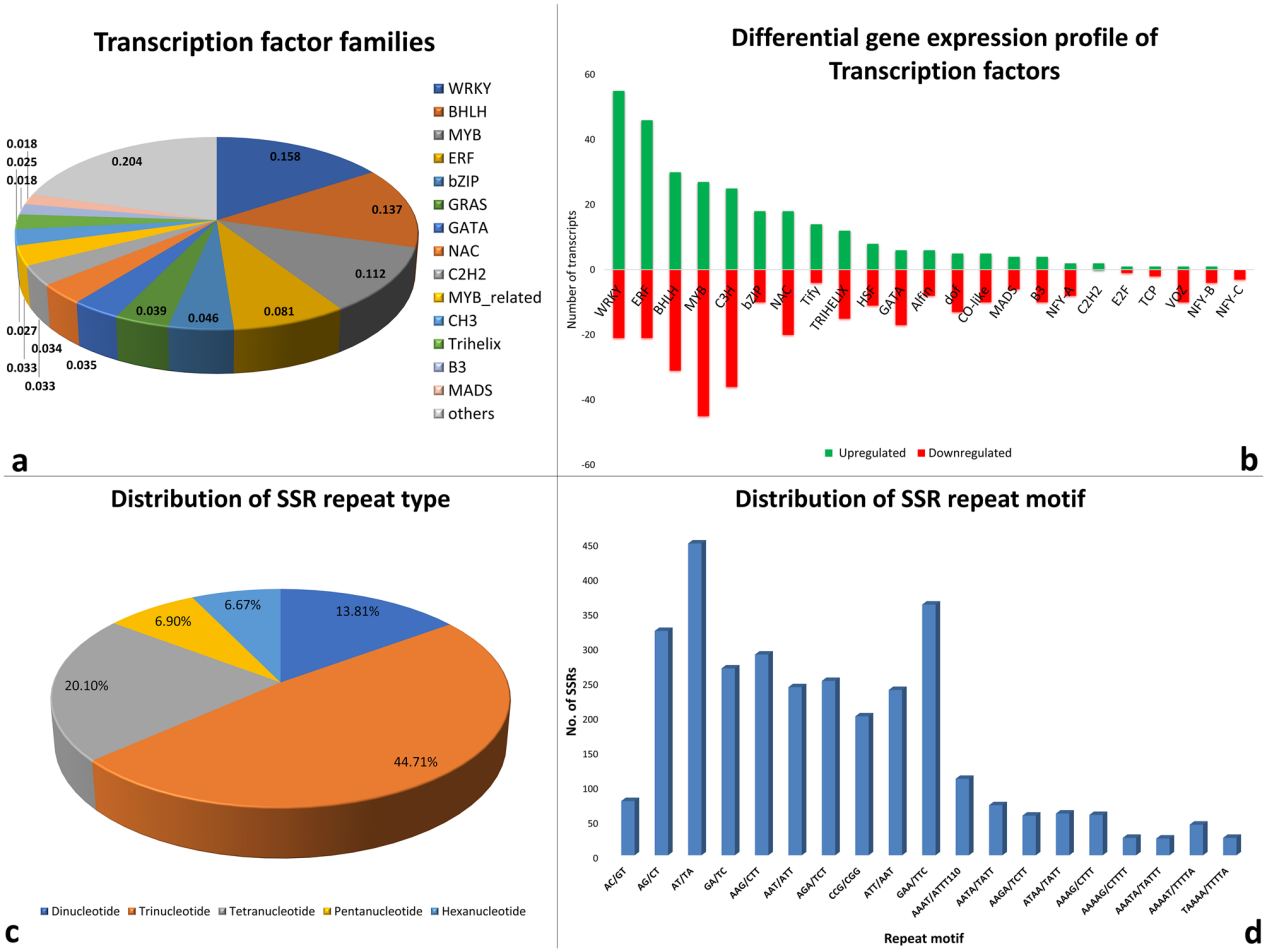
(**a**) Transcription factor families and (**b**) Differential gene expression profile of TFs, (**c**) Distribution of SSR repeat type and (**d**) Distribution of SSR repeat motif.

Transcription factors are sequence-specific DNA binding trans-regulatory protein and are reported to mediate the gene expression with reference to numerous developmental and environmental stimuli by recognizing specific cis-regulatory DNA sequences at the promoter region of their target gene. Moreover, the transcription factors are found to play a significant function in the regulation of several pathways related to secondary metabolism by controlling the metabolic flux and cellular differentiation to a large extent. Here a total of 1079 unigenes encoding putative transcription factors were identified and further grouped into 46 different TFs families in the *O. turpethum* transcriptome, out of which WRKY constitute the most abundant TFs family with 173 unigenes (15.7%) followed by BHLH (150, 13.6%), MYB (123,11.2%), ERF (88, 8.02%), bZIP (50, 4.5%) and GRAS (43, 3.9%) (Fig. 7a). WRKY TF is one of the major and largest groups of plant specific TFs families which are previously reported to be involved in regulating several biological processes such as the development of plant^36^, responses to pathogen entry^37^, nutritional deficiency, endosperm, seed, embryo and micropyle development and senescence^38^, responses to different biotic and abiotic stress, phytohormone signaling pathway and also demonstrated to a play major role in regulating the expression of gene engaged in the biosynthesis of various secondary metabolites like flavanols, phenolic compounds including lignin and tannins^39,40^. Besides WRKY, other TFs families such as bHLH, MYB, C2H are also involved in the secondary metabolism pathway^41^.

Among the TFs encoding unigenes (1097) identified in *O. turpethum* transcriptome*,* 597 unigenes classified in 24 TFs families exhibited differential expression with 291 upregulated unigene in root versus stem comparison. 55 unigenes of WRKY TFs family followed by ERF (46), bHLH (30) was found to be highly upregulated in the stem tissues whereas, the most frequently upregulated genes in root tissues belongs to TFs family MYB (45) followed by C3H (36) and bHLH (31) (Fig. 7b). The high expression level of WRKY transcription factor in stem tissue might be regulating the biosynthesis of terpenoids as reported in the *Gossypium arboreum* and *Taxus chinensis* where GaWRKY1 and TaWRKY1 were found to regulate the synthesis of gossypol and paclitaxel biosynthesis^42,43^.

### Identification of SSR

Simple sequence repeat (SSR) or Microsatellite are stretches of DNA consisting of short tandem repeat motifs of 1–6 nucleotides. SSRs were first applied to plant science by^44^ and over the past 30 years, it has been extensively used in plant genotyping as they are highly reproducible and transferable among related species, informative, multiallelic in nature and exhibit co-dominant inheritance. Basically, the SSR markers are favorable in genetic diversity study, estimation of gene flow and rate of crossing over and construction of linkage map, QTL mapping, the study of genetic relatedness and population structure, cultivar identification, and DNA fingerprinting^45,46^. The emergence of high throughput next-generation sequencing approach has come up with a new framework for identifying microsatellites. Currently there are no studies addressing the genetic diversity and classification of germplasm resources of *O. turpethum* based on SSR markers as they have not been discovered so far. In this study, the genic SSRs are identified for the first time using the largescale transcriptome data which could be helpful for the genetic and breeding studies. For the identification of SSRs, the *O. turpethum* transcripts were searched with MISA software. Of the 64,259 transcripts of *O. turpethum*, a total of 8585 potential SSR loci were discovered. The number of SSRs containing sequences in *O. turpethum* was 6970. The frequency of SSRs is 13.4% and the average distribution distance is 3361 bp (Table 3). The number of transcripts containing more than one SSR was 1248 and the number of SSRs present in compound form was 346, where the maximum number of bases interrupting 2 SSRs in a compound microsatellite is 10. Analysis of the data showed that the most abundant motif type in *O. turpethum* was the trinucleotide repeats (4174:48.30%), also recorded in studies of other plant species such as *I. nil, I. batatas, T. cordifolia, P. ovata*^47–50^. The next class of repeat motifs observed frequently was tetranucleotide repeats (1840), followed by dinucleotide repeat (1320), pentanucleotide (653) and hexanucleotide (614) repeat motifs (Fig. 7c). Among all identified SSRs, the AAG/CTT trinucleotide motif was found to be the most abundant accounting for the largest fraction (11.4%:977) off SSRs followed by AG/CT dinucleotide repeat motif with a frequency of 7.4% (Fig. 7d). This frequency of distribution appears to contradict the previous findings in most plant genomes such as *I. batatas*, *I. nil* where the AG/CT dinucleotide motif repeat was found to be most abundant which may be due to the different genetic makeup of different species and the different standards used for the search of SSRs. Furthermore, the current study reported the occurrence of the CCG/CGG trinucleotide motif, the most predominant motif in monocot plant species, which accounts for 6.4% of total SSRs detected. But the recent findings do not support the notion of the rare appearance of CCG/CGG motif in most dicot plant species.

**Table 3:** SSR statistics.

Results of microsatellite search	Root-Stem
Total number of sequences examined	64,259
Total size of examined sequences (bp)	2,885,611
Total number of identified SSRs	8585
Number of SSR containing sequences	6970
Number of sequences containing more than 1 SSR	1248
Number of SSRs present in compound formation	346

This study reports the discovery of genome-wide SSRs for the first time in *O. turpethum,* which will enrich the molecular marker resource of *O. turpethum* and will also be helpful in further research related to genetic diversity studies, genetic linkage mapping, and marker-assisted selection to trigger the traditional plant breeding.

## Conclusion

Presumably, the current investigation for the first time reported the transcriptome analysis of root and stem tissues of *O. turpethum* which was carried out without any reference genome using Illumina HiSeq 2500 platform. The study generated a total number of 76,790 transcripts and 64,259 unigenes with an average sequence length of 449 bp, pooled from the root and stem tissues together. About 32.5% of the identified unigenes were annotated and functionally classified in 6 databases. The KEGG pathway analysis provides an overview of important pathways along with identification of a large number of candidate genes encoding key enzymes involved in secondary metabolites including phenylpropanoid, flavonoids, and terpenoid biosynthesis. Various conserved transcription factor families were also predicted for the advancement of future genomic research of the plant. Furthermore, a total number of 8585 potential genomic SSRs were identified in this study representing the first report of its type which would be a cost-efficient resource for genetic diversity assessment and marker assisted breeding by developing functional molecular markers. Altogether this finding will further prosper the knowledge on the biosynthesis, regulation, tissue-specific accumulation of important bioactive compounds and their enhancement through genetic engineering along with the selection of superior allele governing the desired trait for breeding of the potential medicinal plant *O. turpethum* in the future. However, further biochemical and pharmacological research is needed in order to reveal the differences between the root and stem tissues of *O. turpethum*.

## Material and methods

### Plant materials and RNA extraction

**Figure 8:**
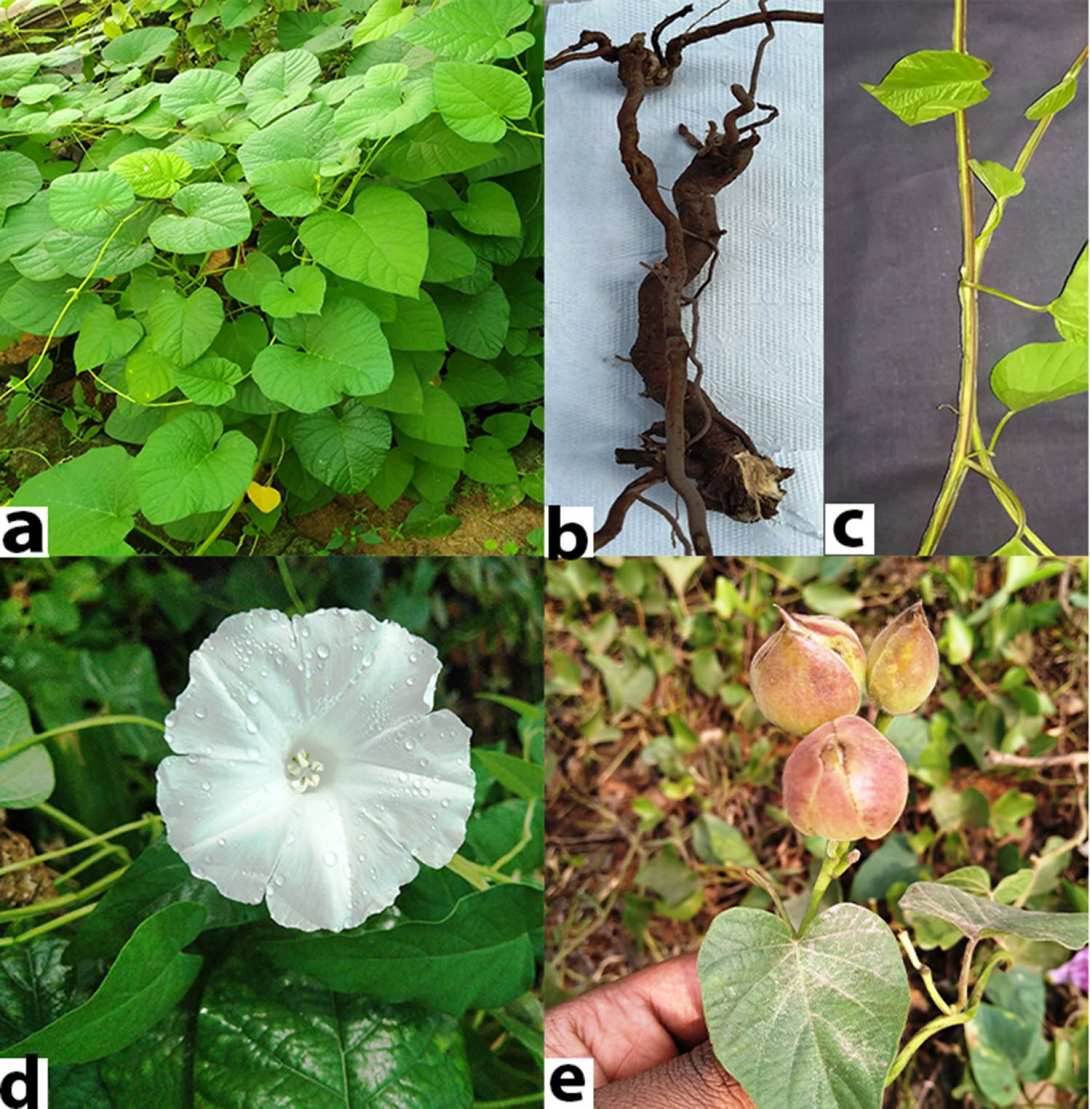
(**a**) O. turpethum plant, (**b**) Root, (**c**) Stem, (**d**) Flower and (**e**) Fruit.

The root and stem of *O. turpethum* (Fig. 8), collected from the local medicinal plant garden (Bhubaneswar, Odisha, India) with requisite permission from the garden administrator. The samples were collected from two different plants and were used as biological replicates. The samples were quick-frozen in liquid nitrogen and subsequently preserved at − 80 °C till further analysis. The plant was taxonomically identified and authenticated by Dr. Laxmikanta Acharya (Associate professor, Centre for Biotechnology, Siksha ‘O’ Anusandhan University, Odisha, India) and a voucher specimen (SOAU/CBT/2020/OP/30) was retained in the department for future reference and the plant has been maintained in an environmentally controlled greenhouse. Experimental research on the plant used for the study complies with relevant institutional, national, and international guidelines and legislation. The total RNA of two tissues (root and stem) of *O. turpethum* was isolated using RNeasy Plant Mini Kit. 1% Formaldehyde Denaturing Agarose gel and Qubit® 2.0 Fluorometer was used for checking the quantity and quality of the isolated RNA.

### Illumina 2 × 150 paired end library preparation and sequencing

The total RNA isolated was subjected to oligo dT magnetic beads for the enrichment of mRNA. The purified mRNA was then subjected to fragmentation at an appropriate temperature. The first strand cDNA of the fragmented mRNA was generated using RT-PCR (Reverse-Transcription PCR) which was then followed by synthesis of the second strand cDNA, A-base addition, adaptor-index ligation and lastly amplification. The libraries of amplified cDNA were screened through HS (High Sensitivity) DNA chip on Bioanalyzer 2100 (Agilent Technologies) according to the instructions provided by manufacturer. After the quality assessment, the library was subjected to 2 × 150 bp PE chemistry on Illumina platform for cluster generation and sequencing. Sequencing of the template from both forward and reverse direction is facilitated by the paired end sequencing on the Illumina 2500 platform.

### De novo assembly and CDS prediction

The raw sequence data acquired from sequencing were subjected to quality control screening which includes elimination of low-quality reads and adaptor or primer sequences containing reads. The clean reads generated after data processing were assembled using Trinity software (Version 2.1.1) with a fixed k-mer size of 25. The non-redundant clustered transcripts (unigenes) were predicted from the assembled transcripts using CD-hit software (version-4.6.1). Finally, all the assembled unigenes of the stem and root tissues were further processed for the prediction of CDS regions using the TransDecoder tool (http://transdecoder.github.io) at default parameters with a minimum length of 100 amino acids of the encoded protein plus homology search with Pfam and UniProt databases.

### Homology search and functional annotation of unigenes

The *O. turpethum* unigenes were searched against NCBI’s “Non-redundant (NR)” database using BLASTX with an E-value cut off of 1E^−5^ to identify the sequence conservation. Further, for the functional characterization, the unigenes were submitted to BLASTX search against Uniport, Pfam, KOG/COG database. The GO mapping of the NR annotated sequences were obtained through Blast2GO command line V-1.4.1. For the assignment of orthologs and prediction of metabolic pathways in *O. turpethum,* the unigenes were compared against the KEGG database through KEGG automatic annotation server (KAAS) using BLASTX with a threshold bit-score value of 60 (default).

### Differential gene expression analysis

Master de novo assembly generated using combined reads of Root and Stem samples was used for DEG (Differentially Expressed Gene) analysis. The reads of Root and Stem samples were mapped separately to unigenes sequences obtained from master assembly using bwa v0.7.12-r1039. Finally, these mapped reads with Root (control) versus Stem (Treated) combination were given as input to DESeq Bioconductor package in R, which consequently provides normalized values regarded as “basemean” which was further utilized for logFC and *p* value evaluation. In-house R-script (Xcelris proprietary Script) was employed to delineate the distribution and graphical representation of differentially expressed genes found in Root-versus-Stem samples. The criteria for the identification of DEGs were described in the table below (Table 4).

**Table 4:** Criteria for the identification of DEGs.

Condition	Status
log2FC > 0	Up regulated
log2FC < 0	Down regulated
log2FC > 0 and *p* value < 0.05	Significantly up regulated
log2FC < 0 and *p* value < 0.05	Significantly down regulated

Further, the Top 50 significantly expressed genes (i.e. highly up and highly downregulated genes) were depicted in form of heatmap through MeV (Multiple Experiment Viewer) using a hierarchical clustering approach.

The complete workflow for Illumina Sequencing de novo Assembly, Annotation, and other Bioinformatics Analysis carried out in the Root- Stem Transcriptome of *O. turpethum* is provided in Supplementary Fig. S9.

### Identification of transcription factor

The transcription factor families were identified by homology searches of the assembled unigenes against the plant transcription factor database (Plant TFDB v5.0) using BLASTX with an *E-value* cut off of 1E^−5^.

### qRT-PCR validation of gene expression profiling

For the experimental validation of gene expression patterns in root and stem tissues of *O. turpethum*, ten candidate genes involved in the phenylpropanoid and terpenoid biosynthesis pathway were randomly selected. The actin gene (CDS_16344_Unigene_52248) was used as an endogenous reference. The gene-specific primers were designed using Primer3plus software and the primer sequences are given in the supplementary file (Supplementary Table S8). The qRT-PCR experiments were performed as described by Mangrauthia et al.^51^. The relative expression levels of the selected candidate genes were normalized to actin gene and calculated using the 2 − ΔΔCt method^52^. All qRT- PCR experiments were carried out in three biological and three technical replications.

### SSR identification

All the assembled unigenes were subjected to SSR detection by using a Perl script program MISA (Microsatellite finder tool) (http://pgrc.ipk-gatersleben.de/misa/misa.html). For search parameter, the minimum number of repetitions for di-, tri-, tetra-, penta-, hexa-nucleotide was set to 6, 5, 3, 3, 3 respectively.

## Supplementary Information

Supplementary Figures.

Supplementary Tables.

## Data Availability

The generated raw sequence data were deposited to NCBI Sequence Read Archive (SRA) database under the Bio Project Accession No PRJNA655823 and SRA accession No SRX8904980 (for root tissue) and SRX8904981 (for stem tissue). The authors confirm that the data supporting the findings of this study are available within the article and its supplementary materials. Any other relevant data are available upon request from the corresponding author.

## Abbreviations

CDS: Coding sequence
NR: Non-redundant
KEGG: Kyoto encyclopedia of genes and genomes
SSR: Short sequence repeats
NGS: Next generation sequencing
GO: Gene ontology
KOG: EuKaryotic orthologous groups
DEG: Differentially expressed gene
bp: Base pair
UV: Ultraviolet
QTL: Quantitative trait loci
