# Supplementary material for: De novo transcriptome and tissue specific expression analysis of genes associated with biosynthesis of secondary metabolites in Operculina turpethum (L.)
Source: Sci Rep. 2021 Nov 18;11:22539. doi: 10.1038/s41598-021-01906-y (PMC8602414; doi:10.1038/s41598-021-01906-y)
Supplement: Supplementary file 1 — Supplementary Figures. [file 41598_2021_1906_MOESM1_ESM.pdf]

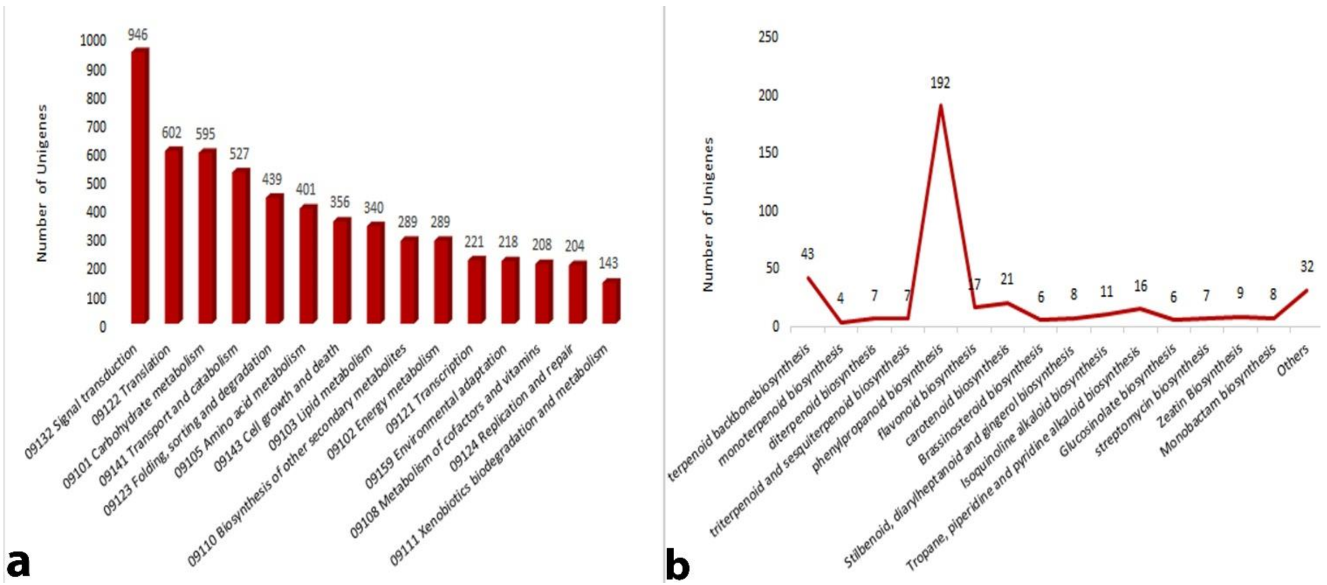

Figure S1: The top 15 largest KEGG pathways

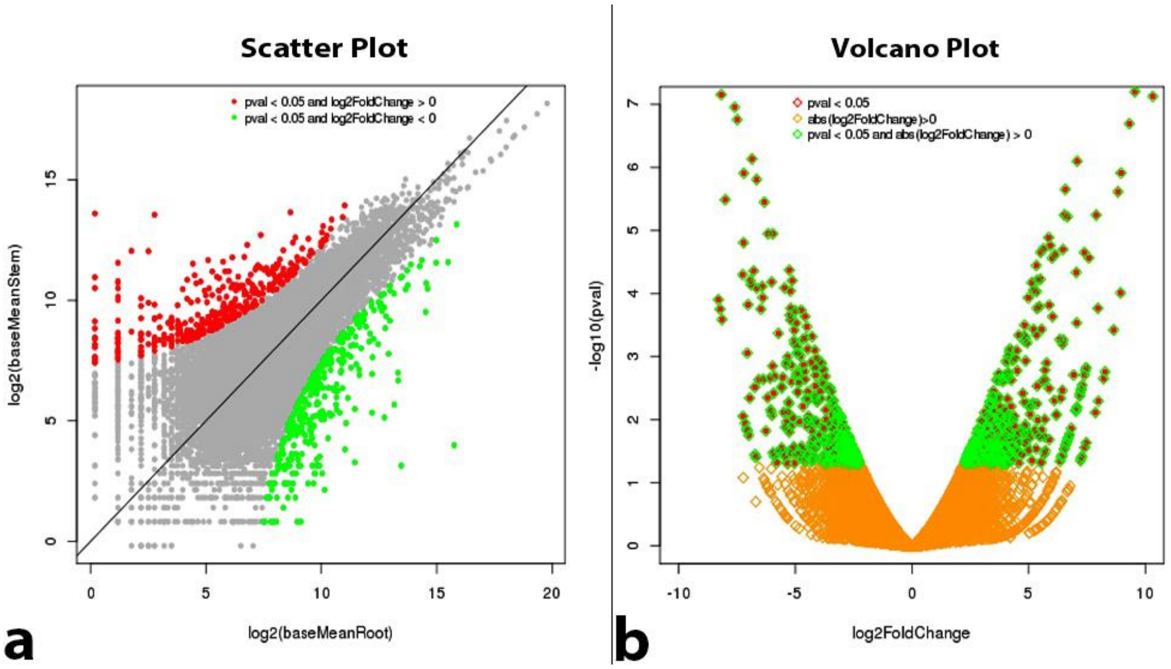

Figure S2: a) Scatter plot for normalized values obtained through DESeq basemean values of all differentially expressed genes in Root-vs-Stem. Each dot indicates one gene. The dots above the black diagonal line indicate up-regulated genes and below this line are down-regulated genes. Where red dots represent significantly upregulated genes with Pvalue<0.05 and log2FC >0 and green dots represents significantly downregulated genes with Pvalue<0.05 and log2FC < 0. The horizontal coordinates represent the log2(basemean)values of Root and the vertical coordinates the Stem log2(basemean) values.

b) Volcano plots of the distribution of gene expression for Root-Vs-Stem samples. DESeq was performed to show the differentially expressed genes. Red, green, and orange correspond to genes with p-value < 0.05, absolute log2FC <0 and (p-value < 0.05 and absolute log2FC >0) respectively.

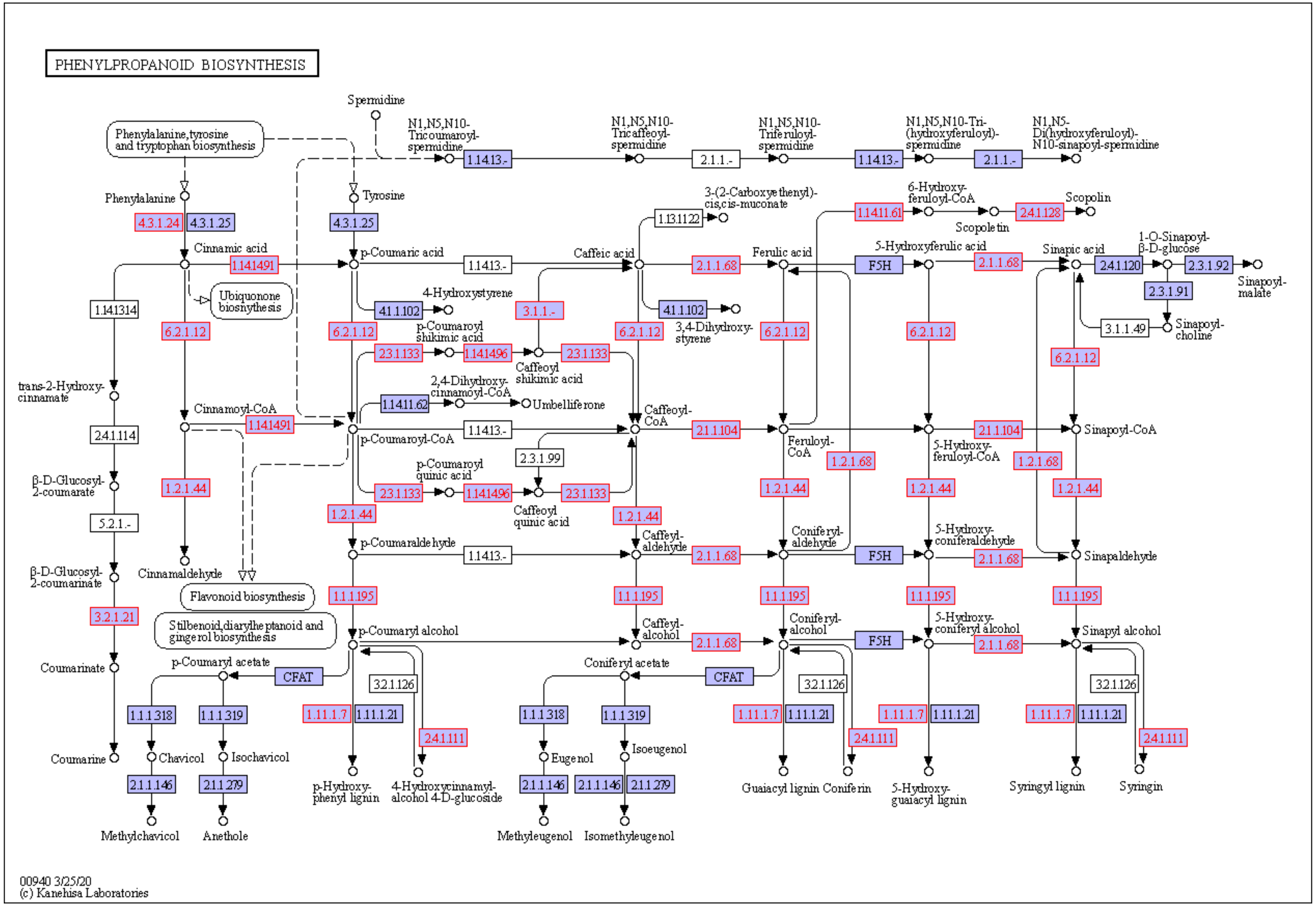

Figure S3. The color-coded map corresponds to map ko00940 in the KEGG database. (https://www.kegg.jp/kegg/pathway.html) The Red-color box represents the unigenes encoding key enzyme involved in Phenylpropanoid Biosynthesis pathway in *O. turpethum.*

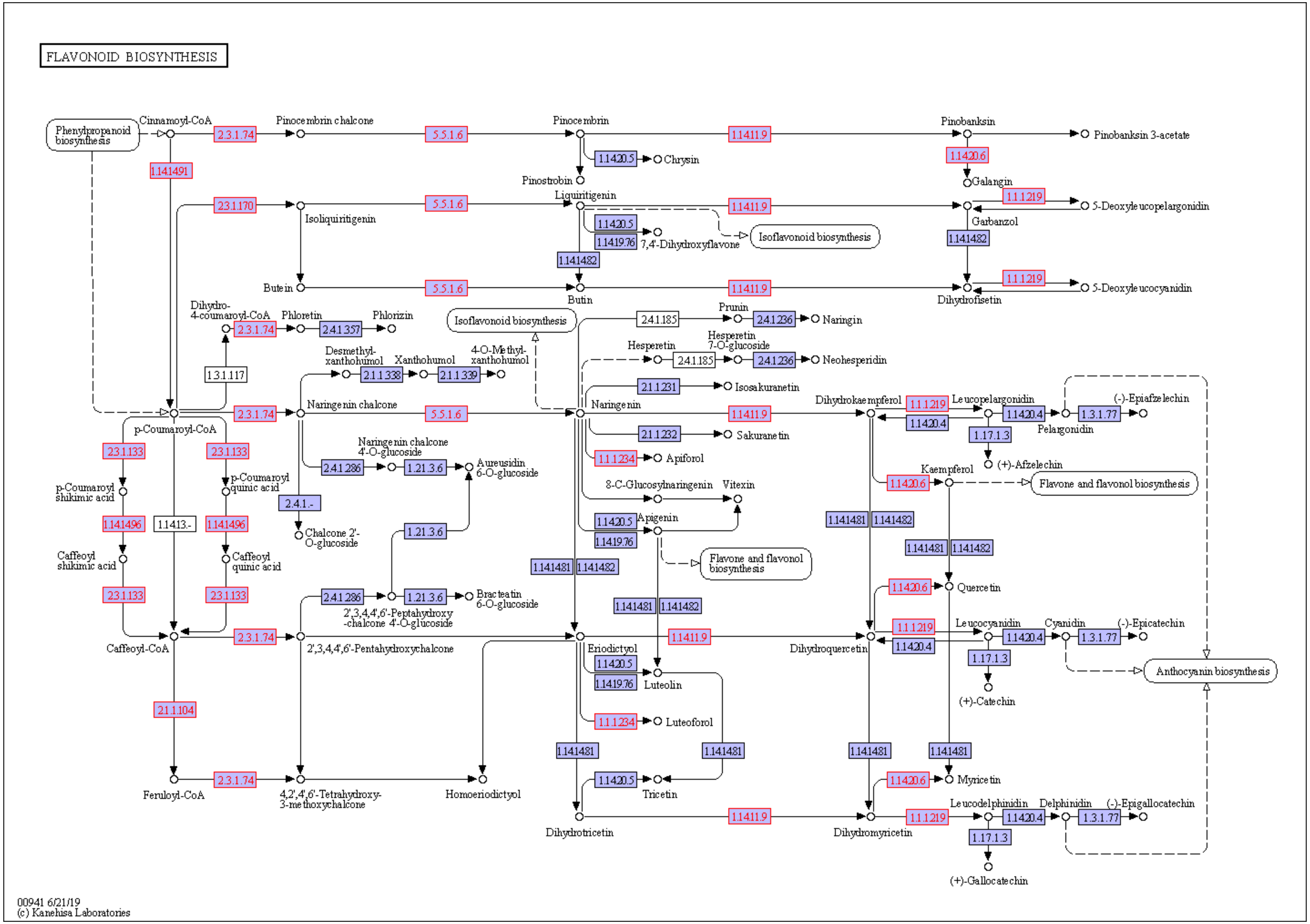

Figure S4. The color-coded map corresponds to map ko00941 in the KEGG database. (https://www.kegg.jp/kegg/pathway.html) The Red-color box represents the unigenes encoding key enzyme involved in Flavonoid Biosynthesis pathway in *O. turpethum.*

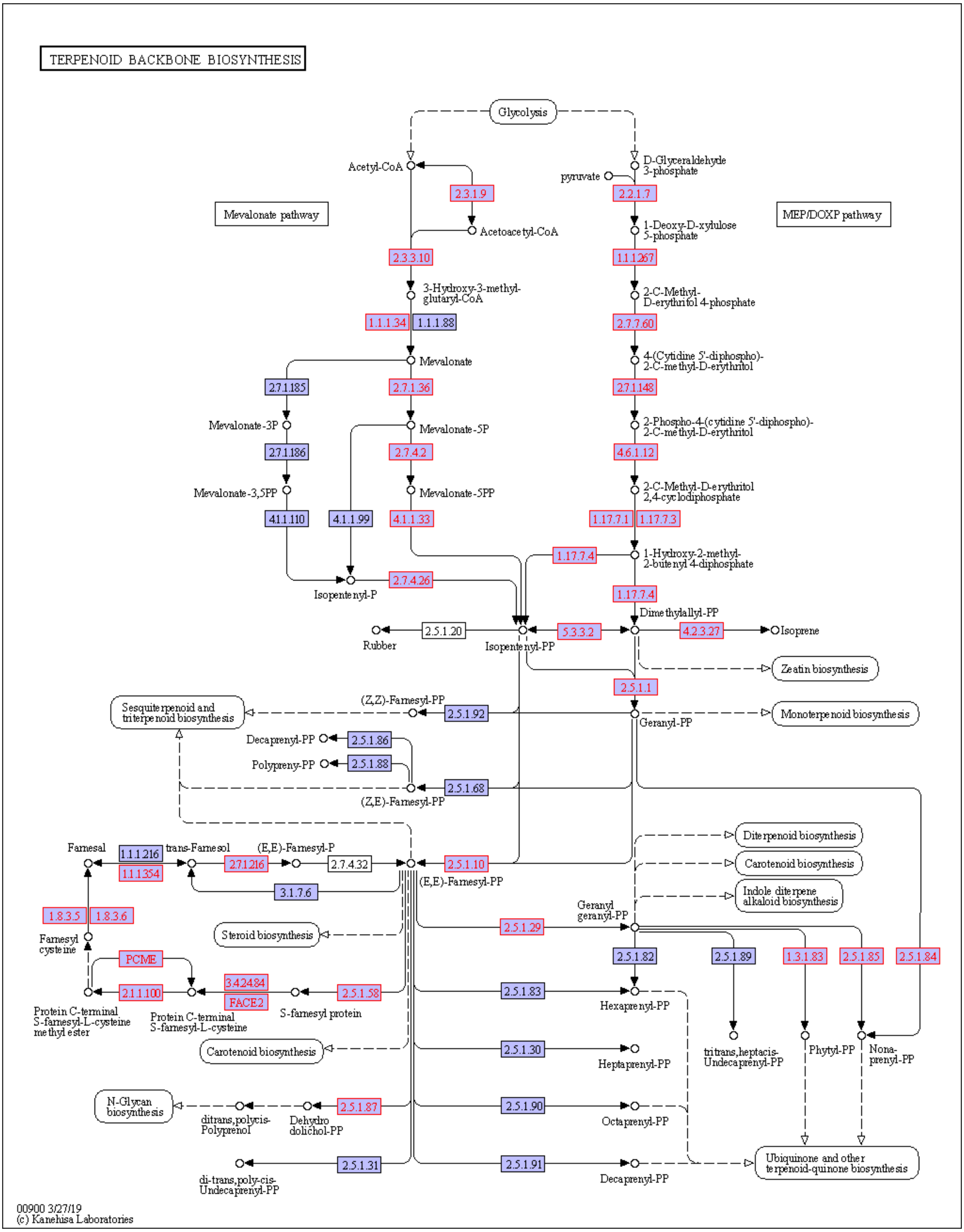

Figure S5. The color-coded map corresponds to map ko00900 in the KEGG database. (https://www.kegg.jp/kegg/pathway.html) The Red-color box represents the unigenes encoding key enzyme involved in Terpenoid backbone Biosynthesis pathway in *O. turpethum.*

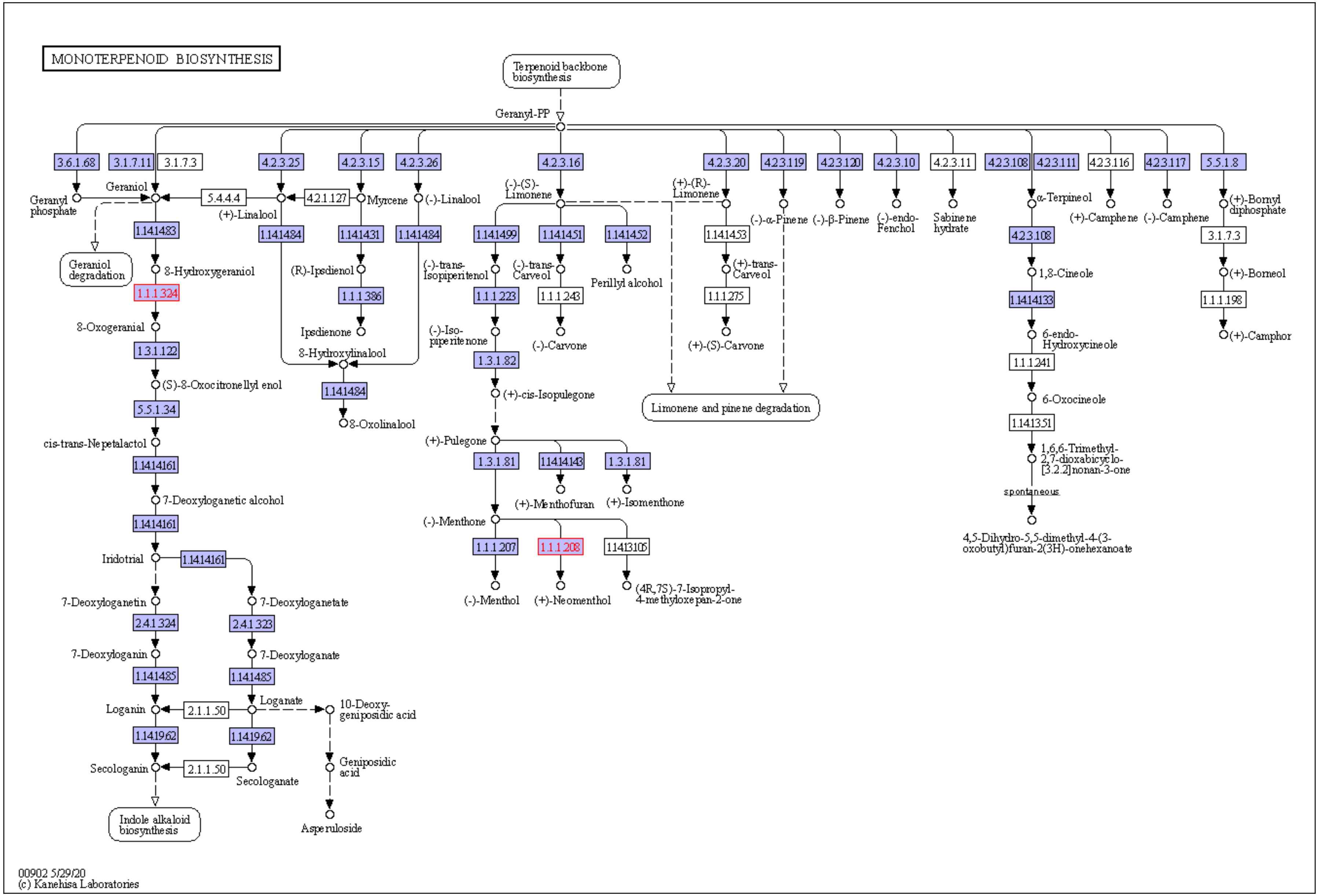

Figure S6. The color-coded map corresponds to map ko00902 in the KEGG database. (https://www.kegg.jp/kegg/pathway.html) The Red-color box represents the unigenes encoding key enzyme involved in Monoterpenoid Biosynthesis pathway in *O. turpethum.*

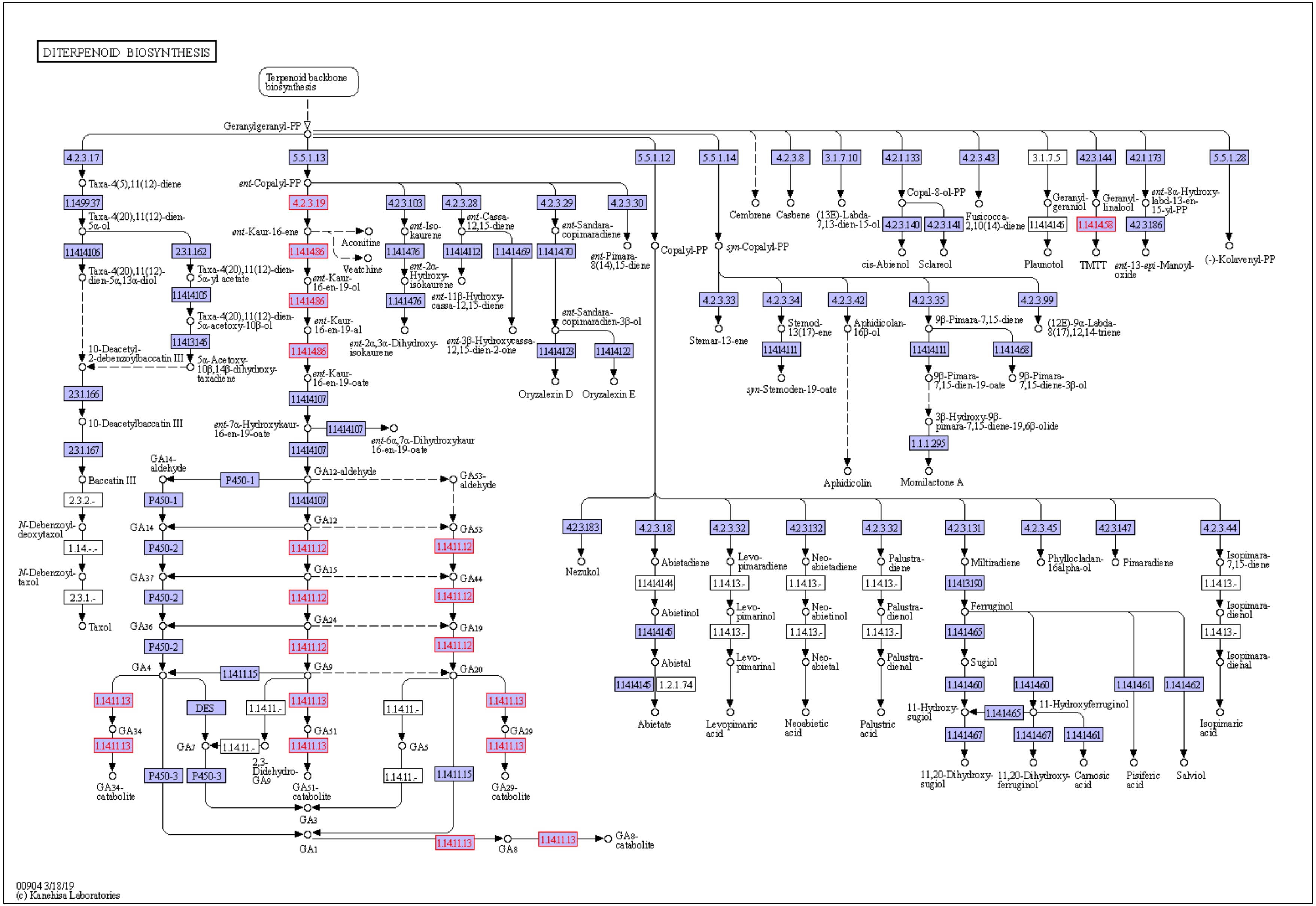

Figure S7. The color-coded map corresponds to map ko00904 in the KEGG database. (https://www.kegg.jp/kegg/pathway.html) The Red-color box represents the unigenes encoding key enzyme involved in Diterpenoid Biosynthesis pathway in *O. turpethum.*

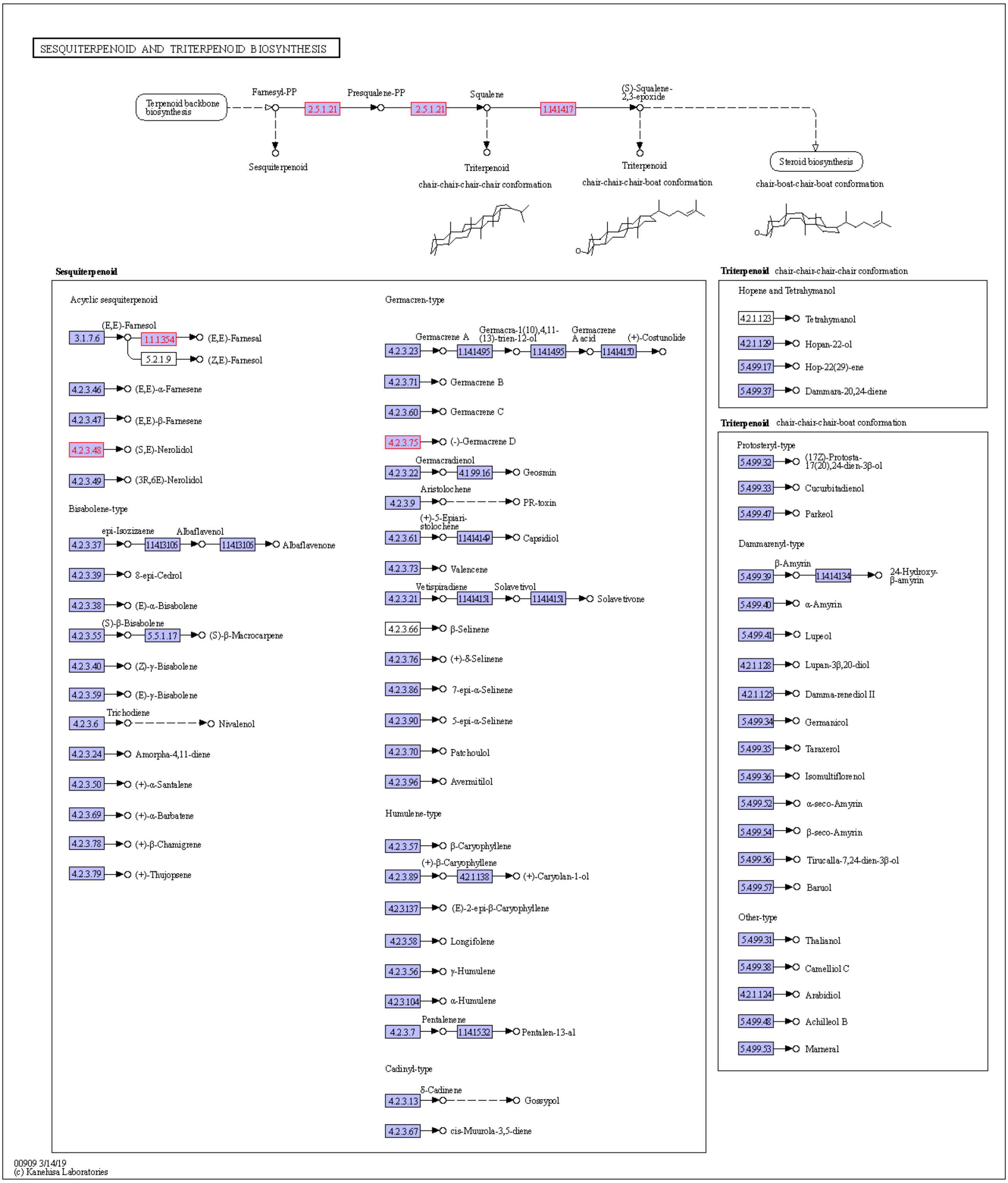

Figure S8. The color-coded map corresponds to map ko00909 in the KEGG database. (https://www.kegg.jp/kegg/pathway.html) The Red-color box represents the unigenes encoding key enzyme involved in Sesquiterpenoid and triterpenoid Biosynthesis pathway in *O. turpethum*.

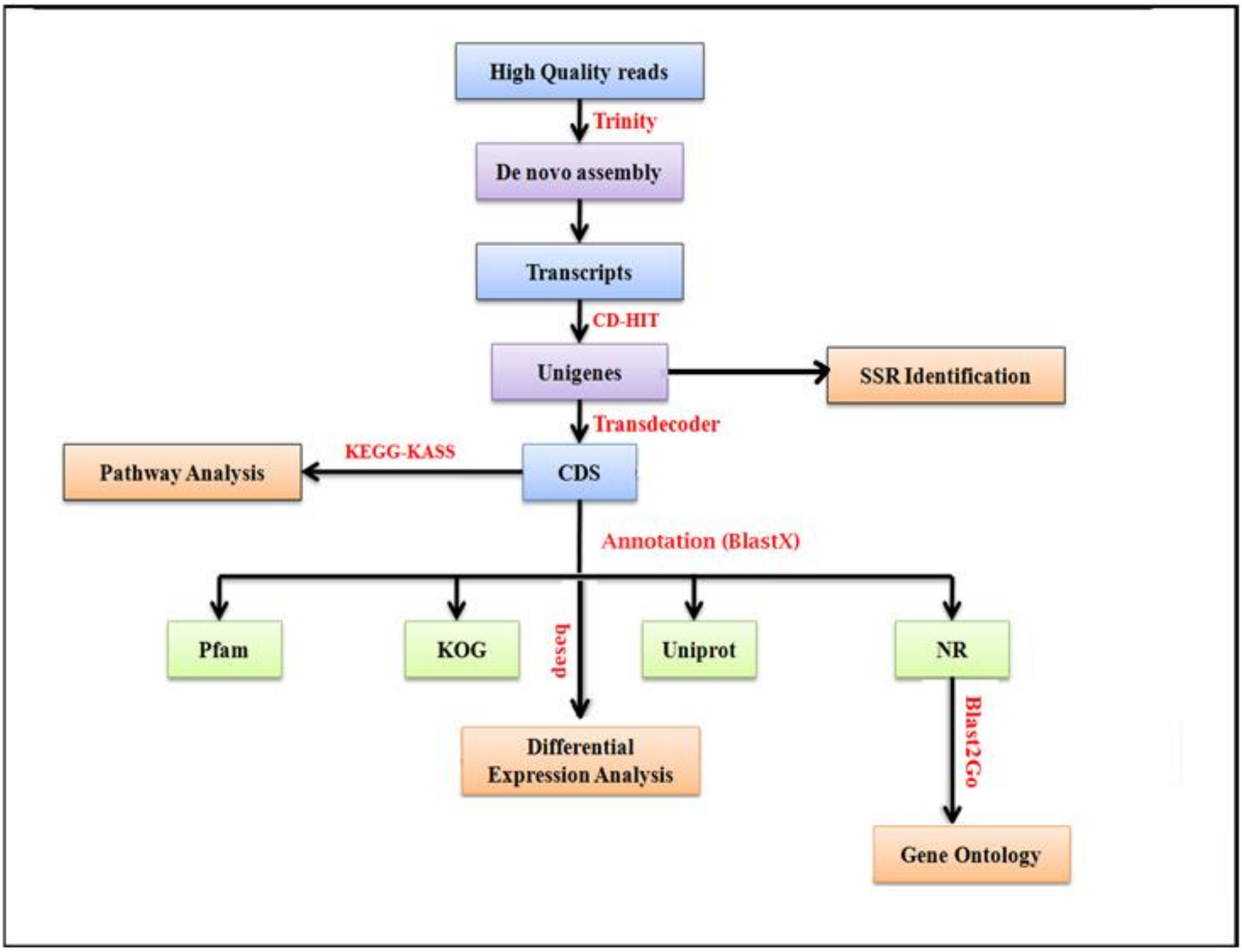

Figure S9. Workflow for Illumina Sequencing de novo Assembly, Annotation and other Bioinformatics Analysis carried out in the Root- Stem Transcriptome of *O. turpethum*.
